# Adverse childhood experiences: a retrospective study to understand their associations with lifetime mental health diagnosis, self-harm or suicide attempt, and current low mental wellbeing in a male Welsh prison population

**DOI:** 10.1186/s40352-020-00115-5

**Published:** 2020-06-12

**Authors:** Kat Ford, Mark A. Bellis, Karen Hughes, Emma R. Barton, Annemarie Newbury

**Affiliations:** 1 Public Health Collaborating Unit, School of Health Sciences, College of Human Sciences, Bangor University, Wrexham, LL13 7YP UK; 2 World Health Organization Collaborating Centre on Investment for Health and Wellbeing, Policy and International Health Directorate, Public Health Wales, Wrexham, LL13 7YP UK

**Keywords:** Adverse childhood experiences, Prison, Incarceration, Mental illness, Mental health, Mental wellbeing, Suicide, Self-harm, Prisoners

## Abstract

**Background:**

Prisoners are at increased risk of poor mental health and self-harming behaviours, with suicide being the leading cause of death in custody. Adverse childhood experiences (ACEs) such as child maltreatment are strong predictors of poor mental health and wellbeing yet despite high levels of ACEs in offender populations, relatively few studies have explored the relationships between ACEs and prisoners’ mental health and wellbeing. We conducted an ACE survey with 468 male adult prisoners in a Welsh prison who were not currently considered to be at risk of self-harm and suicide and explored relationships between ACEs, lifetime mental illness diagnosis, self-harm (lifetime and lifetime in prison) or suicide attempt (lifetime and lifetime in prison), and current low mental wellbeing.

**Results:**

Most participants (84.2%) had suffered at least one ACE and 45.5% had suffered ≥4 ACEs. Prevalence of lifetime mental illness diagnosis, self-harm (lifetime and lifetime in prison) or suicide attempt (lifetime and lifetime in prison), and current low mental wellbeing increased with exposure to ACEs. For example, 2.7% of those with no ACEs reported lifetime self-harm or suicide attempt in prison compared with 31.0% (self-harm in prison) and 18.3% (suicide attempt in prison) of those with ≥4 ACEs. Compared with participants with no ACEs, those with ≥4 ACEs were four times more likely to report lifetime mental illness diagnosis and suicide attempt, and over 10 times more likely to report lifetime self-harm than those with no ACEs. Independent of lifetime mental illness diagnosis, self-harm or suicide attempt, participants with ≥4 ACEs were almost three times more likely to have current low mental wellbeing than those with no ACEs.

**Conclusions:**

Male prisoners that have suffered multiple ACEs are substantially more likely to have lifetime mental illness diagnosis, self-harm or suicide attempt, and to have current low mental wellbeing whilst in prison. Findings suggest that trauma-informed approaches are needed in prisons to support prisoner mental health and wellbeing.

## Background

Prisoner mental health is a worldwide public health concern (Borschmann et al. 2018; Jack et al. 2018). Individuals who are incarcerated are often characterised by complex and multiple health needs and the experience of imprisonment, including isolation, insecurity, and a lack of privacy, can negatively impact an individual’s mental health and wellbeing (Konrad et al. 2007). Mental wellbeing (MWB) relates to how people feel and how well they can cope with day to day life, and has been described as feeling good and functioning well (Regan et al. 2016). Although correlated to mental illness, MWB is an independent concept - individuals may have low MWB in the absence of mental illness, and conversely can have mental illness but high MWB (Taggart et al. 2015). However, low MWB is associated with vulnerability to mental illness alongside poorer health outcomes and behaviours (Howell et al. 2007; Stranges et al. 2014). Studies evidence a higher prevalence of current low MWB, mental illness and self-harm amongst those incarcerated than the general population (Fazel et al. 2016; Kariminia et al. 2007; Tweed et al. 2018, 2019), whilst suicide is the leading cause of death in custody globally (Butler et al. 2018). In England and Wales, rates of self-harm in the male adult prison population have been increasing, with over 43,000 incidents reported in the year ending September 2018; a rate of 540 incidents per 1000 prisoners (Ministry of Justice 2019). There were 89 self-inflicted deaths among male prisoners in 2018; a rate of 1.1 per 1000 prisoners (Ministry of Justice 2019). An investigation into deaths in prison found that while 70% of individuals with a self-inflicted death had been identified as having mental health needs, less than half had had these needs flagged on entry to prison (Prisons and Probation Ombudsman 2016).

While poor mental health is a known risk factor for self-harm and suicidal behaviour in male prisoners, a range of other risk factors have also been identified, such as low educational attainment, homelessness, and being on remand/unsentenced or serving a life sentence (Jenkins et al. 2005; Pope 2018). Further, a growing body of evidence is suggesting that a history of adverse childhood experiences (ACEs) is also a key risk factor (Clements-Nolle et al. 2009; Marzano et al. 2011). The term ACEs is used to describe a range of stressful and potentially traumatic events that children can be exposed to whilst growing up, such as child maltreatment, witnessing domestic violence, parental substance abuse or having a household member incarcerated. Such experiences can impact children’s neurobiological, social and emotional development and increase their risks of health and social harms throughout the life course (Berens et al. 2017). Further, risks of poor life course health outcomes increase along with the number of ACE types suffered, and particularly strong relationships are identified between ACEs and mental illness, self-harm and suicide attempt, as well as behaviours conducive to criminal justice involvement such as violence, problematic drug use, and youth and prolific offending (Baglivio and Epps 2016; Baglivio et al. 2014; Hughes et al. 2017). Accordingly, the prevalence of ACEs has been shown to be substantially higher in justice-involved populations than general populations (Skarupski et al. 2016) and poorer mental health and greater suicidality (Godet-Mardirossian et al. 2011) has been found among prisoners with ACEs. For example, studies with incarcerated males have found increased levels of depression, psychological distress, and personality disorders among those with specific ACE types, such as childhood physical and sexual abuse (Roberts et al. 2008; Sergentanis et al. 2014; Skarupski et al. 2016; Wolff and Caravaca Sánchez 2019; Wolff and Shi 2012; Zhang and Zheng 2018). However, to our knowledge no previous studies have explored associations between exposure to cumulative ACEs and prisoner MWB, while most studies exploring relationships between cumulative ACEs and prisoner mental illness have focused on women. Research in female prison populations has identified that a mental illness diagnosis and suicide attempt are associated with increasing numbers of ACEs (Friestad et al. 2014; Messina and Grella 2006).

Imprisonment provides a unique opportunity to identify and support individuals who may be at risk of mental illness, self-harm and suicide attempt. Developing a better understanding of the association between ACEs, current low MWB and lifetime mental illness diagnosis, self-harm or suicide attempt in the prison population can help to identify where preventative work can be directed. The detection of prisoners at high-risk of these negative outcomes and delivery of appropriate care may also provide an important step in reducing wider health disparities in this population (Borschmann et al. 2018).

## Methods

### Aim

We use primary data collected in a Welsh male prison population (Ford et al. 2019) to explore associations between ACEs and lifetime mental illness diagnosis, self-harm (lifetime and lifetime in prison) or suicide attempt (lifetime and lifetime in prison), and whether ACEs predict current low MWB in prisoners.

### Data collection

A convenience sampling method was used to recruit a sample of incarcerated males in Wales. Her Majesties Prison (HMP) Parc^1^ was selected as the research site due to it housing the largest prison population in Wales at the time of data collection (approximately 1700 in January 2018; Ministry of Justice 2018). Study inclusion criteria were: aged 18–69 years; cognitively able to participate; and not being managed under the care planning process for being at risk of suicide or self-harm (Assessment, Care in Custody & Teamwork [ACCT] procedures; Ministry of Justice 2013). This latter criteria was included as individuals managed under ACCT were considered too vulnerable to participate in the study. To provide an adequate sample size with high ACEs for analyses, we aimed to survey a third of the eligible prison population (estimated to be 1448 at the time of data collection).

The study was advertised through electronic information points on each prison unit in advance of fieldwork and through the distribution of leaflets advertising the study during data collection (February to June 2018). Trained researchers approached potential participants on the prison units during free time, outlining the study aims and methodology. Names and prison identification numbers were taken for study volunteers and a suitable time/date was arranged for them to complete the interview (Monday-Thursday, 8:00 am-6:30 pm). At the point of interview, the researcher verbally summarised and provided participants with a study information sheet outlining: the study purpose and voluntary, anonymous, and confidential nature; their right to withdraw; that participation or a decline to participate would not affect their care; and a contact within the prison for any issues or complaints. Participants were given the opportunity to ask questions and provided written informed consent before proceeding with the survey. Face-to-face interviews were undertaken by researchers who completed questionnaires using pen and paper, with participants given the opportunity to self-complete the more sensitive questions (e.g. ACE questions). Following survey completion, participants were provided with a thank you leaflet including contact details for a designated lead within the prison and information on available help and support accessible within the prison. No personal identifiable data were collected during the interview, with the exception of the written record of consent, which was stored separately to the study questionnaire. All study materials were available in English and Welsh and respondents could complete the survey in either language.

During the study period, contact was made with 696 individuals, of whom 188 (27.0%) declined participation and 508 volunteered to take part. Of the individuals who opted to participate in the study, 12 (2.4%) were ineligible and 26 (5.2%) left the prison before they were able to participate. Of the 658 eligible individuals remaining in prison that were approached for participation, 470 individuals completed the questionnaire, resulting in a study participation rate of 71.4%. However, any individuals who did not provide all ACE data required for analysis (*n* = 2) were removed from the sample, resulting in a final sample of 468.

### Questionnaire / measures

All questionnaire measures were self-reported. Standardised ACE survey questions from the US Centers for Disease Control and Prevention short ACE tool (Centers for Disease Control and Prevention n.d.) and the World Health Organization’s Short Child Maltreatment Questionnaire (Meinck et al. 2016) were used to measure exposure to 11 categories of ACE (before 18 years of age: 1) physical abuse, 2) verbal abuse, 3) sexual abuse, 4) emotional neglect, 5) physical neglect, 6) parental separation, 7) witnessing domestic violence, and living with a household member who was 8) a problem alcohol user, 9) a drug user, 10) mentally ill, or 11) incarcerated; see Additional file 1: Table S1). The ACE tool has been validated as a reliable tool for the retrospective assessment of ACEs (Meinck et al. 2016). In line with previous studies (Bellis et al. 2015; Felitti et al. 1998), the number of ACEs reported by participants was summed (possible range 0–11 ACEs) and categorised into an ACE count variable (0 ACEs, 1 ACE, 2–3 ACEs, and ≥ 4 ACEs).

To measure lifetime mental illness diagnosis, participants were asked if they had ever been diagnosed by a doctor or nurse with any mental health condition (e.g. depression, anxiety or other mental illness), using a question adapted from the national Welsh ACE survey (Hughes et al. 2018).

Using questions taken from the Measuring the Quality of Prison Life (MQPL) questionnaire routinely used in UK prisons (Liebling et al. 2011), participants were asked to report lifetime (1) self-harm or (2) suicide attempt, with response options: *no, never attempted; yes, outside of prison only; yes, in prison only;* and, *yes, outside and in prison,* Responses to self-harm and suicide attempt questions were dichotomised ‘no’ and ‘yes’ for four outcomes: ‘lifetime self-harm’, ‘lifetime suicide attempt’, ‘lifetime self-harm in prison’ and ‘lifetime suicide attempt in prison’.

Current MWB was measured using the validated Short Warwick-Edinburgh Mental Wellbeing Scale (SWEMWBS; Stewart-Brown et al. 2009), which asks individuals how often over the past 2 weeks they have been: *feeling optimistic about the future; feeling useful; feeling relaxed; dealing with problems well; thinking clearly; feeling close to other people; able to make up their own mind about things*. Responses are scored using a Likert scale: 1, none of the time; 2, rarely; 3, some of the time; 4, often; 5, all of the time). An overall MWB score was calculated by summing response scores for the seven questions and transforming total raw scores to metric score in line with guidelines (possible range 7 [lowest] to 35 [highest]; Stewart-Brown et al. 2009). Current low MWB was categorised as scores ≤19.59 based on cut offs from general population level data in Wales (Davies et al. 2019; low scores > 1 standard deviation [SD] below the mean). Responses to the seven individual components of SWEMWBS were also dichotomised to indicate low ratings (never or rarely in the last 2 weeks; yes or no).

Participant demographic information collected included age, ethnicity (self-identified using UK census categories) and education qualifications attained (none; secondary school or equivalent [level 2]; college or equivalent [level 3]; university or equivalent [≥level 4]; based on the National Qualifications Framework/Qualification and Credit Framework). Age was categorised into four groups (18–20; 21–29; 30–39; and ≥ 40 years) and ethnicity was re-categorised into *White* and *other* due to low numbers in individual non-White ethnic groups.

### Statistical analysis

Data entry and statistical analyses were completed using SPSS v24. Analyses employed cross-tabulations and chi-square tests to initially examine bivariate associations between ACEs, demographic characteristics, and the study outcomes of interest: lifetime mental illness diagnosis, self-harm (lifetime and lifetime in prison) or suicide attempt (lifetime and lifetime in prison), current low MWB, and low ratings for the seven individual SWEMWBS components. Logistic regression was then employed to examine the independent contributions of ACEs on each outcome of interest, controlling for demographics (i.e. adjusted for age, ethnicity and qualifications). A further logistic regression model was used to explore the associations between ACEs and current low MWB controlling for demographics, lifetime mental illness diagnosis, self-harm or suicide attempt.

## Results

### Sample characteristics

Over half (54.9%) of the men in the sample were aged 30 years and over, with less than one in ten (9.2%) aged 18–20 (Table 1). The majority (84.2%) reported White ethnicity and almost a third (31.0%) reported having no educational qualifications. Most participants (84.2%) reported having been exposed to at least one ACE, with just under half (45.5%) reporting exposure to ≥4 ACEs.

**Table 1:** Sample characteristics and prevalence of lifetime mental illness diagnosis, low MWB and self-harm and suicide outcomes

Category		All	Percentage reporting outcome					
			Lifetime mental illness diagnosis	Self-harm		Suicide attempt		Current Low MWB^**a**^
				Lifetime	Lifetime in prison	Lifetime	Lifetime in prison	
**All**	%		48.7	33.1	19.9	32.1	10.7	29.6
**ACE count**	0	15.8	35.1	8.1	2.7	17.6	2.7	13.5
	1	17.7	33.7	18.1	10.8	13.3	3.6	24.1
	2–3	20.9	37.8	31.6	16.3	25.5	6.1	22.4
	≥4	45.5	64.3	48.4	31.0	47.4	18.3	40.8
	*X*^2^		38.388	51.807	35.246	45.615	24.405	25.401
	*P*		< 0.001	< 0.001	< 0.001	< 0.001	< 0.001	< 0.001
**Age group (years)**	18–20	9.2	27.9	25.6	14.0	25.6	4.7	25.6
	21–24	16.2	43.4	42.1	32.9	28.9	10.5	25.3
	25–29	19.7	45.7	29.3	16.3	33.7	17.4	29.3
	30–39	28.0	53.4	33.6	17.6	30.5	7.6	28.2
	≥40	26.9	56.3	32.5	19.0	36.5	11.1	35.2
	*X*^2^		12.758	4.496	10.271	2.564	7.281	2.987
	*P*		0.013	0.343	0.036	0.633	0.122	0.560
**Ethnicity**	White	84.2	52.5	35.0	21.3	34.8	11.9	31.8
	Other	15.8	28.4	23.0	12.2	17.6	4.1	17.8
	*X*^2^		14.555	4.086	3.281	8.467	4.049	5.788
	*P*		< 0.001	0.043	0.070	0.004	0.044	0.016
**Qualifications**	No qualifications	31.0	49.0	30.3	18.6	33.1	9.7	37.2
	Secondary school	36.5	48.5	40.4	27.5	35.1	12.3	30.0
	College/6th form	25.0	50.4	30.8	14.5	30.8	12.0	23.3
	Higher education	7.5	42.9	17.1	5.7	17.1	2.9	17.1
	*X*^2^		0.624	8.866	12.87	4.458	3.066	8.906
	*P*		0.891	0.031	0.005	0.216	0.382	0.031

*ACE* Adverse childhood experience; *MWB* Mental wellbeing

^a^Scores ≤19.59

Nearly half (48.7%) of participants reported having lifetime mental illness diagnosis, and almost a third reported lifetime self-harm (33.1%) or suicide attempt (32.1%; Table 1). In total 40.4% of the sample reported lifetime self-harm or suicide attempt, and 21.4% lifetime self-harm or suicide attempt whilst in prison. Of individuals reporting self-harm, 60.0% reported lifetime self-harm in prison (19.9% of all participants) and 28.4% had only ever self-harmed in prison (9.4% of all participants). Of those reporting suicide attempt, 33.3% reported lifetime suicide attempt in prison (10.7% of all participants) and 18.0% had only ever attempted-suicide in prison (5.8% of all participants).

Three in ten (29.6%) participants were categorised as having current low MWB. The proportion responding low ratings (i.e. ‘never’ or ‘rarely’ in the last 2 weeks) to the seven individual components of SWEMWBS ranged from 7.1% for *able to make up my own mind about things*, to 33.6% for *feeling close to others* (Table 2).

**Table 2:** Prevalence of low individual components of SWEMWBS by demographics and ACE count

		% in the last 2 weeks that have never or rarely been						
		Feeling optimistic	Feeling useful	Feeling relaxed	Dealing with problems well	Thinking clearly	Feeling close to others	Able to make up my own mind
**n**		467	466	467	467	467	467	467
**All**	%	17.3	27.3	25.7	16.7	14.3	33.6	7.1
**ACE count**	0	14.9	17.6	9.5	6.8	4.1	14.9	4.1
	1	14.5	26.5	21.7	12.0	8.4	26.5	4.8
	2–3	16.3	27.6	16.3	14.3	12.2	28.6	9.2
	≥4	19.8	30.8	37.3	23.1	21.2	45.3	8.0
	*X*^2^	1.770	4.872	30.281	13.227	17.259	27.587	2.623
		0.621	0.181	< 0.001	0.004	0.001	< 0.001	0.454
**Age group (years)**	18–20	11.6	37.2	18.6	14.0	16.3	27.9	7.0
	21–24	16.0	29.3	25.3	17.3	13.3	29.3	6.7
	25–29	19.6	23.9	29.3	18.5	18.5	31.5	7.6
	30–39	16.0	27.5	24.4	14.5	14.5	38.2	6.9
	≥40	19.8	24.8	27.0	18.3	11.1	34.9	7.1
	*X*^2^	2.097	3.214	2.000	1.137	2.547	2.738	0.690
	*P*	0.718	0.523	0.736	0.888	0.636	0.603	0.999
**Ethnicity**	White	19.5	28.2	26.6	17.8	15.5	33.5	8.1
	Other	5.5	21.9	20.5	11.0	8.2	34.2	1.4
	*X*^2^	8.497	1.243	1.201	2.052	2.644	0.015	4.276
	*P*	0.004	0.265	0.273	0.152	0.104	0.902	0.039
**Qualifications**	No qualifications	20.7	31.0	29.0	21.4	19.3	33.8	10.3
	Secondary school	18.7	25.9	25.1	15.8	12.3	31.6	5.8
	College/6th form	12.9	27.6	24.1	14.7	15.5	36.2	5.2
	Higher education	11.4	17.1	20.0	8.6	0.0	34.3	5.7
	*X*^2^	3.786	3.018	1.581	4.395	9.493	0.676	3.491
	*P*	0.286	0.389	0.664	0.222	0.023	0.879	0.322

*ACE* Adverse childhood experience, *SWEMWBS* Short Warwick-Edinburgh Mental Wellbeing Scale

### Bivariate analysis

In chi-squared analysis, levels of lifetime mental illness diagnosis, self-harm or suicide attempt (lifetime and lifetime in prison), and current low MWB were significantly higher in White participants (Table 1). Lifetime self-harm (lifetime and lifetime in prison) and current low MWB were associated with low levels of educational attainment (Table 1). The prevalence of lifetime mental illness diagnosis increased with age (*p* = 0.013), whilst lifetime self-harm in prison was highest amongst those aged 21–24 (*p* = 0.036).

All outcomes explored were strongly associated with ACE count (*p* < 0.001, Table 1). Lifetime mental illness diagnosis almost doubled from 35.1% in those with no ACEs to 64.3% in those with ≥4 ACEs. Similar increases were seen for all self-harm and suicide attempt outcomes, with levels of lifetime self-harm in prison increasing from 2.7% in those with no ACEs to 31.0% in those with ≥4 ACEs and levels of lifetime suicide attempt in prison increasing from 2.7% to 18.3% respectively. The proportion of respondents with current low MWB tripled from 13.5% in those with no ACEs to 40.8% in those with ≥4 ACEs (Table 1).

The proportions reporting low ratings for the individual SWEMWBS components: *feeling relaxed, dealing with problems well, thinking clearly* and *feeling close to others* also increased significantly with ACE count (Table 2).

### Multivariate analysis

Logistic regression analyses explored the independent relationships between ACEs and lifetime mental illness diagnosis, self-harm (lifetime and lifetime in prison) or suicide attempt (lifetime and lifetime in prison), controlling for relationships with demographics. High ACEs remained strongly related to each of these outcomes (Table 3). Compared with those with no ACEs, those with ≥4 ACEs were around four times more likely to have lifetime mental illness diagnosis (adjusted odds ratio [AOR] 3.96, *p* < 0.001) and to have lifetime suicide attempt (AOR 4.36, *p* < 0.001), and eight times (AOR 7.98, *p* = 0.005) more likely to have lifetime suicide attempt in prison. There were no associations between lower levels of ACEs and the outcomes: lifetime mental illness diagnosis and suicide attempt. However, odds of lifetime self-harm (lifetime and lifetime in prison) were substantially elevated in those with both ≥4 ACEs and 2–3 ACEs. Compared with those with no ACEs, those with ≥4 ACEs were over ten times (AOR 10.7, *p* < 0.001) more likely to have lifetime self-harm and 15 times (AOR 15.1, *p* < 0.001) more likely to have lifetime self-harm in prison. Independent relationships were also found between White ethnicity and lifetime mental illness diagnosis, self-harm (lifetime and lifetime in prison) or suicide attempt (lifetime and lifetime in prison); between lifetime self-harm in prison and lower education qualifications; and between lifetime mental illness diagnosis and older age (Table 3).

**Table 3:** Adjusted odds ratios (AORs) for lifetime mental illness diagnosis, self-harm and suicide attempt by demographics and ACE count

	Lifetime mental illness diagnosis			Self-harm						Suicide attempt					
				Lifetime			Lifetime in prison			Lifetime			Lifetime in prison		
	AOR	95% CIs	*P*	AOR	95% CIs	*P*	AOR	95% CIs	*P*	AOR	95% CIs	*P*	AOR	95% CIs	*P*
**Age group (years)**															
18–20	0.31	0.14–0.68	0.004	0.77	0.33–1.77	0.530	0.76	0.27–2.11	0.597	0.63	0.28–1.45	0.276	0.45	0.10–2.17	0.322
21–24	0.56	0.30–1.05	0.069	1.43	0.75–2.73	0.280	2.05	1.01–4.18	0.047	0.64	0.33–1.24	0.183	0.86	0.33–2.24	0.758
25–29	0.64	0.36–1.15	0.133	0.78	0.41–1.46	0.431	0.73	0.34–1.55	0.408	0.84	0.46–1.56	0.587	1.72	0.76–3.90	0.192
30–39	0.92	0.54–1.56	0.751	0.96	0.54–1.70	0.893	0.84	0.43–1.67	0.623	0.71	0.41–1.25	0.238	0.63	0.26–1.53	0.308
40+	Ref		0.024	Ref		0.463	Ref		0.056	Ref		0.615	Ref		0.161
**Qualifications**															
No qualifications	1.10	0.49–2.50	0.813	1.30	0.47–3.58	0.618	2.16	0.47–10.08	0.325	1.78	0.65–4.88	0.263	2.39	0.29–19.97	0.421
Secondary school	1.00	0.45–2.24	0.993	2.18	0.80–5.91	0.126	3.88	0.86–17.64	0.079	1.99	0.73–5.39	0.176	3.10	0.38–25.29	0.291
College/6th form	1.21	0.53–2.77	0.657	1.52	0.54–4.27	0.429	1.74	0.36–8.36	0.489	1.77	0.64–4.94	0.274	3.59	0.43–30.12	0.239
Higher education	Ref		0.907	Ref		0.143	Ref		0.029	Ref		0.604	Ref		0.563
**Ethnicity**															
White	3.18	1.71–5.69	< 0.001	2.22	1.19–4.13	0.012	2.39	1.09–5.24	0.030	2.82	1.45–5.49	0.002	3.29	0.97–11.5	0.056
**ACE count**															
0	Ref		< 0.001	Ref		< 0.001	Ref		< 0.001	Ref		< 0.001	Ref		< 0.001
1	1.05	0.53–2.08	0.887	2.48	0.90–6.85	0.080	3.96	0.81–19.28	0.089	0.71	0.29–1.72	0.444	1.27	0.20–7.94	0.797
2–3	1.22	0.63–2.34	0.555	5.56	2.15–14.39	< 0.001	7.00	1.53–32.07	0.012	1.66	0.77–3.59	0.194	2.52	0.49–13.05	0.272
≥ 4	3.96	2.20–7.11	< 0.001	10.69	4.39–26.04	< 0.001	15.08	3.54–64.27	< 0.001	4.36	2.22–8.56	< 0.001	7.98	1.85–34.42	0.005

Reference category for ethnicity = other; *ACE* Adverse childhood experience, *Ref* Reference category

Logistic regression analysis was also used to explore relationships between ACEs and current low MWB (Table 4). A first model controlling for demographics found that individuals with ≥4 ACEs were four times (AOR 4.35, *p* < 0.001) more likely to have current low MWB than those with no ACEs. There were no associations at lower ACE counts.

**Table 4:** Adjusted odds ratios (AORs) for current low mental wellbeing (MWB; Scores ≤19.59)

	Demographics and ACE count only			Including historical mental health measures		
		Model 1			Model 2	
	AOR	95% CIs	*P*	AOR	95% CIs	*P*
**Age group (years)**						
18–20	0.58	0.26–1.32	0.195	0.73	0.31–1.72	0.475
21–24	0.56	0.29–1.10	0.090	0.57	0.28–1.16	0.119
25–29	0.70	0.38–1.29	0.250	0.77	0.41–1.46	0.430
30–39	0.74	0.42–1.30	0.297	0.77	0.43–1.38	0.378
40+	Ref		0.446	Ref		0.635
**Qualifications**						
No qualifications	2.44	0.91–6.56	0.078	2.48	0.90–6.86	0.080
Secondary school	1.71	0.64–4.59	0.284	1.62	0.59–4.47	0.348
College/6th form	1.29	0.46–3.60	0.624	1.22	0.43–3.50	0.708
Higher education	Ref		0.930	Ref		0.071
**Ethnicity**						
White	2.20	1.13–4.26	0.020	1.65	0.83–3.28	0.155
**ACE count**						
0	Ref		< 0.001	Ref		0.033
1	2.00	0.85–4.68	0.110	1.97	0.82–4.73	0.129
2–3	1.74	0.76–4.01	0.193	1.50	0.63–3.55	0.361
≥ 4	4.35	2.08–9.08	< 0.001	2.75	1.27–5.98	0.010
**Lifetime mental illness diagnosis**				2.21	1.33–3.65	0.002
**Lifetime self-harm**				1.63	0.91–2.95	0.104
**Lifetime suicide attempt**				1.22	0.68–2.22	0.506

Model 1 includes demographics and ACE count, model 2 includes demographics, ACE count, lifetime mental illness, self-harm or suicide attempt. Reference category for ethnicity = other; *ACE* Adverse childhood experience, *Ref* Reference category

Given the strong relationships identified between ACEs and lifetime mental illness diagnosis, self-harm or suicide attempt (Table 3), a second model was run to explore the association between ACEs and current low MWB controlling for demographics, lifetime mental illness diagnosis, self-harm or suicide attempt. Independent of these lifetime outcomes, having ≥4 ACEs remained predictive of experiencing current low MWB, with participants almost three times more likely to have current low MWB than those with no ACEs (AOR 2.75, *p* = 0.010; Table 4). In this model, participants with lifetime mental illness diagnoses were twice as likely to have current low MWB (AOR 2.21, *p* = 0.002). However, lifetime self-harm or suicide attempt was not found to significantly increase the risk of current low MWB.

Logistic regression was also run to explore relationships between ACEs and low ratings for the seven individual components of SWEMWBS, controlling for demographics. ACEs were associated with low ratings for all individual SWEMWBS components except *feeling optimistic* or *being able to make up my own mind about things* (Table 5).

**Table 5:** Adjusted odds for individual components of SWEMWBS by demographics and ACE count

	Feeling optimistic			Feeling useful			Feeling relaxed			Dealing with problems well			Thinking clearly			Feeling close to others			Able to make up my own mind		
	AOR	95% CIs	*P*	AOR	95% CIs	*P*	AOR	95% CIs	*P*	AOR	95% CIs	*P*	AOR	95% CIs	*P*	AOR	95% CIs	*P*	AOR	95% CIs	*P*
**Ethnicity**																					
White	4.09	1.44–11.64	0.008	1.44	0.78–2.65	0.243	1.44	0.76–2.71	0.264	1.71	0.77–3.80	0.186	2.15	0.87–5.31	0.099	1.01	0.58–1.75	0.975	6.18	0.82–46.28	0.076
**ACE count**																					
0	Ref		0.700	Ref		0.223	Ref		< 0.001	Ref		0.009	Ref		0.002	Ref		< 0.001	Ref		0.521
1	0.91	0.37–2.25	0.837	1.67	0.76–3.66	0.198	2.66	1.03–6.83	0.043	1.79	0.58–5.56	0.313	2.09	0.51–8.54	0.307	2.29	1.01–5.17	0.046	1.13	0.24–5.32	0.880
2–3	1.05	0.45–2.48	0.903	1.71	0.80–3.64	0.166	1.85	0.72–4.81	0.204	2.17	0.73–6.38	0.161	3.07	0.82–11.51	0.096	2.43	1.11–5.33	0.027	2.17	0.55–8.49	0.266
≥ 4	1.32	0.63–2.79	0.463	2.05	1.04–4.05	0.038	5.78	2.50–13.36	< 0.001	3.94	1.49–10.44	0.006	6.13	1.82–20.68	0.003	5.29	2.60–10.78	< 0.001	2.02	0.56–7.27	0.281

Age and education were also entered into the model but were not significant for any outcome. Reference category for ethnicity = other; *ACE* Adverse childhood experience, *Ref* Reference category

## Discussion

Improving the mental health and wellbeing of prisoners is a complex task and it is essential that the risk factors for poor mental health and wellbeing in prison are understood (Phillips et al. 2018). This study has aimed to identify associations between ACEs and lifetime mental illness diagnosis, self-harm or suicide attempt in incarcerated males, and to explore if ACEs predict current low MWB in prison. Levels of ACE exposure reported by participants were substantially higher than those measured in the Welsh general population, with 84.4% of male prisoners reporting at least one of the 11 ACEs measured and 45.5% reporting ≥4 ACEs; compared with 46.3% (at least one ACE) and 11.9% (≥4 ACEs) respectively in males in the general population (Hughes et al. 2018). Consistent with other international studies (Butler et al. 2018), reported levels of lifetime mental illness diagnoses, self-harm or suicide attempt among prisoners were also elevated compared to general population levels (Hughes et al. 2018). Despite our study excluding those that were currently being managed due to risk of self-harm or suicide attempt, one in five participants reported lifetime self-harm whilst in prison and one in ten reported lifetime suicide attempt.

Prisoners that reported multiple ACEs had substantially higher odds of having a history of poor mental health. For lifetime mental illness diagnosis and suicide attempt (lifetime and lifetime in prison), increased risks were seen in those with ≥4 ACEs, while for lifetime self-harm (lifetime and lifetime in prison), odds were higher and elevated even in those with ≥2 ACEs. Critically, individuals with ≥4 ACEs were 15 times more likely to have lifetime self-harm and eight times more likely to have lifetime suicide attempt in prison. An increasing body of evidence is identifying how chronic early life stress can lead to lasting structural changes in the developing brain that can embed vulnerability to poor mental health, affecting aspects including stress responses, coping skills, attachment, and emotional regulation and functioning (Pechtel and Pizzagalli 2011; Teicher et al. 2016). These effects may not only increase offenders’ vulnerability to developing poor mental health in prison, but also their ability to adapt to the prison environment (Skarupski et al. 2016). For example, the reduction of privacy or restrained movement; uncertainty and lack of personal control; social isolation; and aggression or threat of violence in prison may pose additional risk for vulnerable populations, and individuals that have suffered ACEs may suffer re-traumatisation (Crisanti and Frueh 2011; Krammer et al. 2018; Marzano et al. 2011; Welfare and Hollin 2015). Thus the experience of entering prison may compound the effect of ACEs on mental health and wellbeing.

Though widely explored at the population level, few studies have measured MWB in prisoners (Tweed et al. 2018, 2019) and there is a limited evidence base on the factors associated with current low MWB in this group. We found multiple ACEs to be predictive of current low MWB in incarcerated males, even after controlling for lifetime mental illness diagnosis, self-harm or suicide attempt. For the seven individual components of SWEMWBS measured, having ≥4 ACEs was associated with never or rarely (in the last 2 weeks) feeling useful, relaxed, thinking clearly or dealing with problems well, while having one ACE was associated with never or rarely feeling close to others. In particular, this latter component of MWB may impact on prisoners’ ability to form supportive relationships and seek help in the prison setting. Elsewhere, increased ACE count has been shown to be associated with lower levels of perceived social support among offender populations (Krammer et al. 2018) while in the general population, ACEs have been associated with perceiving services as less supportive (Hughes et al. 2018).

Here, primary data collection has generated a novel dataset to examine a number of mental health and wellbeing outcomes in a male English and Welsh prisoner population. Previous work exploring the relationship between exposure to cumulative ACEs, mental illness and suicide attempt has predominantly been conducted within female prison populations (Friestad et al. 2014; Messina and Grella 2006). Although in this manuscript we do not aim to provide a gender comparison, this would be a useful focus for future research to explore.

Attention is increasingly being drawn to the importance of the provision of trauma-informed services and the need for staff to be understanding of the underlying causes behind current low MWB, self-harm and suicidal behaviour (Baglivio and Epps 2016; Krammer et al. 2018; Marzano et al. 2011). Such examples of trauma-informed interventions in the prison setting are starting to emerge (see Biddle et al. 2018). Recent years have also seen an increase in calls for the implementation of routine enquiry to proactively identify ACEs in a variety of health and other settings. Existing screening procedures in prisons are thought to fail to adequately assess and record individuals’ risk of self-harm and suicide (House of Commons Library 2017) and understanding prisoners’ childhood experiences might help identify those who are more vulnerable to low MWB, self-harm, and suicide attempt and direct support services to those at risk of harm. However, the evidence base for routine ACE enquiry is still in its infancy (Ford et al. 2019) and its place within the criminal justice system has yet to be fully explored, Further, consideration of the implementation of enquiry for ACEs within the criminal justice service requires further exploration of the support systems needed to appropriately respond to any disclosures without re-traumatising the individual (Leitch 2017). The potential to use existing personal data held on prisoners to understand risks of low MWB, self-harm or suicide attempt following exposure to ACEs for this purpose also needs exploration.

With projections for further growth of an already high prison rate in England and Wales (House of Commons Library 2019; Walmsley 2018), the burden on prison health care systems placed by self-harm and suicide attempt is likely to also increase (Borschmann et al. 2018). Incarceration has been thought of as a time to focus interventions (Friestad et al. 2014), and work to address the specific mental health and other needs of prisoners with ACEs is likely to not only benefit prison mental health but also support prisoners’ rehabilitation, help build their trust in support services and have broader societal and public health benefits. Thus ACEs have been associated with recidivism in offender populations (Craig et al. 2017), while levels of self-harm and suicide after release from prison are also markedly higher than rates found in the general population (Binswanger et al. 2007; Borschmann et al. 2016). The identification of interventions that can work to support mental health and wellbeing in prisoners affected by ACEs, and of factors that can protect against mental health difficulties in this vulnerable population, are important areas for future research. A focus should also remain on the primary prevention of ACEs. Preventing future generations from being exposed to ACEs and supporting children affected by them, including the families of prisoners, should help reduce risks of offending and criminal justice system involvement in future generations.

### Limitations

There are a number of study limitations which should be recognised in the interpretation of findings. First, a convenience sample was used and therefore the sample cannot be considered to be representative of the prisoners in the prison studied, nor the wider prison population in England and Wales. However, recruitment aimed to maximise the inclusion of all eligible prisoners, and achieved a high participation rate (71.4%). Our definition of suicide attempt did not rely on suicidal intent and no qualitative data was explored on motivations for either of the self-harm or suicide outcomes explored here. However, self-harm and suicide attempt are commonly used as proxies for suicide (Marzano et al. 2009). Self-harm may not be indicative of suicide attempt and therefore self-harm and suicide attempt were examined as distinct behaviours. Equally, medical diagnosis of depression or other mental health conditions is not necessarily an accurate measure of need for services (Martin et al. 2015), and is likely to provide an under-estimate of lifetime mental illness. Further, we explored lifetime measures for mental illness diagnosis, self-harm and suicide attempt, and we did not collect information on the timing of individual outcomes, and consequently could not explore temporal relationships between ACE exposure, the development of mental illness and timings of self-harm and suicide attempt within or outside the prison setting. This is an important area for future research.

As no information was recorded on the individuals who declined participation in the study, we are unable to identify any bias through self-selection to participate. In line with the ACE methodology, ACE data are retrospective and therefore subject to recall-bias. All data were self-reported and due to the sensitive nature of the ACE, self-harm and suicide attempt questions the responses to these items could be subject to reporting accuracy as disclosure of these issues can be stigmatising. The under-reporting of self-harm and suicidal behaviours could contribute to more conservative findings. Further, prisoners who were on an ACCT, by definition those being at risk of self-harm or suicide at the time of interview, were excluded from participation. Nonetheless, the prevalence of both ACEs, self-harm, and suicide as identified here are similar to those identified in other research studies within the prisoner population (Reavis et al. 2013; Skarupski et al. 2016). Finally, while White ethnicity was associated with most outcomes explored here (see Results), there were too few individuals from other ethnicities to explore whether relationships between ACEs and these outcomes varied by ethnicity, and this would be a useful area for further study.

## Conclusions

International evidence has highlighted the detrimental impact that ACEs can have on mental health across the life course (Bellis et al. 2015; Felitti et al. 1998; Hughes et al. 2017). Our study evidences this effect in a UK prison population, showing that prisoners with multiple ACEs are substantially more likely to have a history of mental illness and self-harming behaviour, including self-harm and suicide attempt within prison settings. It also shows that prisoners with multiple ACEs are at risk of current low MWB whilst in prison. Thus findings suggest that prisoners with multiple ACEs may be particularly vulnerable to poor mental health whilst incarcerated, and that prisons may provide a critical opportunity for providing support to this vulnerable population. Thus, policy and interventions to support mental health and wellbeing within prisons should include ensuring that prison staff are trauma-informed and have an understanding of the underlying causes behind these behaviours (Baglivio and Epps 2016; Krammer et al. 2018; Marzano et al. 2011; Ramluggun 2013). Improving the mental health and wellbeing of prisoners is a complex task, but one which is essential to reducing reoffending, improving the health of prisoners and is also likely to benefit wider population public health.

## Supplementary information

**Additional file 1: Table S1.** Questions used to identify ACEs with qualifying responses.

## Data Availability

The datasets analysed during the current study are available from the corresponding author on reasonable request.

## Abbreviations

ACCT: Assessment, Care in Custody and Teamwork
ACEs: Adverse childhood experiences
AOR: Adjusted odds ratio
CI: Confidence interval
HMP: Her Majesties Prison
MWB: Mental wellbeing
MQPL: Measuring the Quality of Prison Life
Ref: Reference category
SD: Standard deviation
SWEMWBS: Short Warwick-Edinburgh Mental Wellbeing Scale

## Footnotes

1 HMP Parc is a Category B Male Prison, for prisoners for whom escape must be made very difficult but the very highest conditions of security are not necessary (House of Commons Library 2015).

## References

[CR1] Baglivio MT, Epps N (2016). The interrelatedness of adverse childhood experiences among high-risk juvenile offenders. Youth Violence and Juvenile Justice.

[CR2] Baglivio MT, Jackowski K, Greenwald MA, Howell J (2014). Serious, violent, and chronic juvenile offenders: a statewide analysis of prevalence and prediction of subsequent recidivism using risk and protective factors. Criminology & Public Policy.

[CR3] Bellis MA, Ashton K, Hughes K, Ford K, Bishop J, Paranjothy S (2015). Adverse childhood experiences and their impact on health-harming behaviours in the Welsh adult population.

[CR4] Berens AE, Jensen SKG, Nelson CA (2017). Biological embedding of childhood adversity: from physiological mechanisms to clinical implications. BMC Medicine.

[CR5] Biddle P, Dyer W, Hand R, Strinati C (2018). Reflections on a project to prevent suicide and self-harm among prisoners identified as high risk in two prisons in northern England. Health & Justice.

[CR6] Binswanger IA, Stern MF, Deyo RA, Heagerty PJ, Cheadle A, Elmore JG, Koepsell TD (2007). Release from prison - a high risk of death for former inmates. New England Journal of Medicine.

[CR7] Borschmann R, Thomas E, Moran P, Carroll M, Heffernan E, Spittal MJ (2016). Self-harm following release from prison: a prospective data linkage study. Australian & New Zealand Journal of Psychiatry.

[CR8] Borschmann R, Young JT, Moran PA, Spittal MJ, Kinner SA (2018). Self-harm in the criminal justice system: a public health opportunity. The Lancet Public Health.

[CR9] Butler A, Young JT, Kinner SA, Borschmann R (2018). Self-harm and suicidal behaviour among incarcerated adults in the Australian Capital Territory. BMC Health & Justice.

[CR10] Centers for Disease Control and Prevention. (n.d.). Behavioral Risk Factor Surveillance System ACE Data. Retrieved from https://www.cdc.gov/violenceprevention/childabuseandneglect/acestudy/ace-brfss.html. Accessed 20 Feb 2020.

[CR11] Clements-Nolle K, Wolden M, Bargmann-Losche J (2009). Childhood trauma and risk for past and future suicide attempts among women in prison. Women's Health Issues.

[CR12] Craig JM, Baglivio MT, Wolff KT, Piquero AR, Epps N (2017). Do social bonds buffer the impact of adverse childhood experiences on reoffending?. Youth Violence and Juvenile Justice.

[CR13] Crisanti AS, Frueh BC (2011). Risk of trauma exposure among persons with mental illness in jails and prisons: what do we really know?. Current Opinion in Psychiatry.

[CR14] Davies AR, Sharp CA, Homolova L, Bellis MA (2019). Population health in a digital age: the use of digital technology to support and monitor health in Wales.

[CR15] Fazel S, Hayes AJ, Bartellas K, Clerici M, Trestman R (2016). Mental health of prisoners: prevalence, adverse outcomes, and interventions. The Lancet Psychiatry.

[CR16] Felitti VJ, Anda RF, Nordenberg D, Williamson DF, Spitz AM, Edwards V (1998). Relationship of childhood abuse and household dysfunction to many of the leading causes of death in adults. American Journal of Preventive Medicine.

[CR17] Ford K, Barton ER, Newbury A, Hughes K, Bezeczky Z, Roderick J, Bellis MA (2019). Understanding the prevalence of adverse childhood experiences (ACEs) in a male offender population in Wales: the prisoner ACE survey.

[CR18] Friestad C, Ase-Bente R, Kjelsberg E (2014). Adverse childhood experiences among women prisoners: relationships to suicide attempts and drug abuse. The International Journal of Social Psychiatry.

[CR19] Godet-Mardirossian, H., Jehel, L., & Falissard, B. (2011). Suicidality in male prisoners: influence of childhood adversity mediated by dimensions of personality. *Journal of Forensic Sciences, 56*, 942–949. 10.1111/j.1556-4029.2011.01754.x. 10.1111/j.1556-4029.2011.01754.x 21447076

[CR20] House of Commons Library (2015). Categorisation of prisoners in the UK: Briefing paper. Number 07437.

[CR21] House of Commons Library (2017). Mental health in prisons: Eighth Report of Session 2017–19.

[CR22] House of Commons Library (2019). UK Prison Population Statistics. Briefing paper. Number CBP-04334.

[CR23] Howell RT, Kern ML, Lyubomirksy S (2007). Health benefits: meta-analytically determining the impact of well-being on objective health outcomes. Health Psychology Review.

[CR24] Hughes K, Bellis MA, Hardcastle KA, Sethi D, Butchart A, Mikton C (2017). The impact of multiple adverse childhood experiences on health: a systematic review and meta-analysis. The Lancet Public Health.

[CR25] Hughes K, Ford K, Davies AR, Homolova L, Bellis MA (2018). Sources of resilience and their moderating relationships with harms from adverse childhood experiences.

[CR26] Jack HE, Fricchione G, Chibanda D, Thornicroft G, Machando D, Kidia K (2018). Mental health of incarcerated people: a global call to action. The Lancet Psychiatry.

[CR27] Jenkins R, Bhugra D, Meltzer H, Singleton N, Bebbington P, Brugha T (2005). Psychiatric and social aspects of suicidal behaviour in prisons. Psychological Medicine.

[CR28] Kariminia A, Butler TG, Corben SP, Levy MH, Grant L, Kaldor JM, Law MG (2007). Extreme cause-specific mortality in a cohort of adult prisoners - 1988 to 2002: a data-linkage study. International Journal of Epidemiology.

[CR29] Konrad N, Daigle MS, Daniel AE, Dear GE, Frottier P, Hayes LM (2007). Preventing suicide in prisons, part I. Crisis.

[CR30] Krammer S, Eisenbarth H, Hügli D, Liebrenz M, Kuwert P (2018). The relationship between childhood traumatic events, social support, and mental health problems in prisoners. The Journal of Forensic Psychiatry & Psychology.

[CR31] Leitch L (2017). Action steps using ACEs and trauma-informed care: a resilience model. BMC Health & Justice.

[CR32] Liebling A, Hulley S, Crewe B, Gadd D, Karstedt S, Messner S (2011). Conceptualising and measuring the quality of prison life. The sage handbook of criminological research methods.

[CR33] Martin MS, Eljdupovic G, McKenzie K, Colman I (2015). Risk of violence by inmates with childhood trauma and mental health needs. Law and Human Behavior.

[CR34] Marzano L, Hawton K, Rivline A, Fazel S (2011). Psychosocial influences on prisoner suicide: a case-control study of near-lethal self-harm in women prisoners. Social Science & Medicine.

[CR35] Marzano L, Rivlin A, Fazel S, Hawton K (2009). Interviewing survivors of near-lethal self-harm: a novel approach for investigating suicide amongst prisoners. Journal of Forensic and Legal Medicine.

[CR36] Meinck F, Steinert JI, Sethi D, Gilbert R, Bellis MA, Mikton C (2016). Measuring and monitoring national prevalence of child maltreatment: a practical handbook.

[CR37] Messina N, Grella C (2006). Childhood trauma and women’s health outcomes in a California prison population. American Journal of Public Health.

[CR38] Ministry of Justice (2013). Management of prisoners at risk of harm to self, to others and from others (Safer Custody).

[CR39] Ministry of Justice (2018). Population bulletin: monthly January 2018.

[CR40] Ministry of Justice (2019). Safety in custody statistics, England and Wales: deaths in prison custody to December 2018, assaults and self-harm to September 2018.

[CR41] Pechtel P, Pizzagalli DA (2011). Effects of early life stress on cognitive and affective function: an integrated review of human literature. Psychopharmacology.

[CR42] Phillips J, Padfield N, Gelsthorpe L (2018). Suicide and community justice. BMC Health & Justice.

[CR43] Pope L (2018). Self-harm by adult men in prison: a rapid evidence assessment (REA). Her majesty’s prison and probation service.

[CR44] Prisons and Probation Ombudsman (2016). Learning from PPO investigations: prisoner mental health.

[CR45] Ramluggun P (2013). A critical exploration of the management of self-harm in a male custodial setting: qualitative findings of a comparative analysis of prison staff views on self-harm. Journal of Forensic Nursing.

[CR46] Reavis JA, Looman J, Franco KA, Rojas B (2013). Adverse childhood experiences and adult criminality: how long must we live before we possess our own lives?. The Permanente Journal.

[CR47] Regan M, Elliott I, Goldie I (2016). Better mental health for all: a public health approach to mental health improvement.

[CR48] Roberts A, Yang M, Zhang T, Coid J (2008). Personality disorder, temperament, and childhood adversity: findings from a cohort of prisoners in England and Wales. The Journal of Forensic Psychiatry & Psychology.

[CR49] Sergentanis TN, Sakelliadis EI, Vlachodimitropoulos D, Goutas N, Sergentanis IN, Spiliopoulou CA, Papadodima SA (2014). Does history of childhood maltreatment make a difference in prison? A hierarchical approach on early family events and personality traits. Psychiatry Research.

[CR50] Skarupski KA, Parisi JM, Thorpe R, Tanner E, Gross D (2016). The association of adverse childhood experiences with mid-life depressive symptoms and quality of life among incarcerated males: exploring multiple mediation. Aging & Mental Health.

[CR51] Stewart-Brown S, Tennant A, Tennant R, Platt S, Parkinson J, Weich S (2009). Internal construct validity of the Warwick-Edinburgh Mental Well-being Scale (WEMWBS): a Rasch analysis using data from the Scottish Health Education Population Survey. Health and Quality of Life Outcomes.

[CR52] Stranges S, Samaraweera PC, Taggart F, Kandala N, Stewart-Brown S (2014). Major health-related behaviours and mental well-being in the general population: the health survey for England. BMJ Open.

[CR53] Taggart F, Stewart-Brown S, Parkinson J (2015). Warwick-Edinburgh Mental Well-being Scale (WEMWBS). User Guide – Version 2.

[CR54] Teicher MH, Samson JA, Anderson CM, Ohashi K (2016). The effects of childhood maltreatment on brain structure, function and connectivity. Nature Reviews Neuroscience.

[CR55] Tweed EJ, Gounari X, Graham L (2018). Mental wellbeing in prisoners in Scotland: an analysis of repeat cross-sectional surveys. Lancet.

[CR56] Tweed, E. J., Gounari, X., & Graham, L. (2019). Mental wellbeing among people in prison in Scotland: an analysis of repeat cross-sectional surveys. *Journal of Public Health*, fdz106. 10.1093/pubmed/fdz106. 10.1093/pubmed/fdz106 PMC8185554 31583401

[CR57] Walmsley R (2018). World prison population list.

[CR58] Welfare HR, Hollin CR (2015). Childhood and offense-related trauma in young people imprisoned in England and Wales for murder and other acts of serious violence: a descriptive study. Journal of Aggression, Maltreatment & Trauma.

[CR59] Wolff N, Caravaca Sánchez F (2019). Associations among psychological distress, adverse childhood experiences, social support, and resilience in incarcerate men. Criminal Justice and Behavior.

[CR60] Wolff N, Shi J (2012). Childhood and adult trauma experiences of incarcerated persons and their relationship to adult behavioral health problems and treatment. International Journal of Environmental Research and Public Health.

[CR61] Zhang J, Zheng Y (2018). Childhood maltreatment profiles among incarcerated Chinese males and their associations with personality disorder symptoms and criminal behaviors. Psychiatry Research.

