# Supplementary material for: Adverse childhood experiences: a retrospective study to understand their associations with lifetime mental health diagnosis, self-harm or suicide attempt, and current low mental wellbeing in a male Welsh prison population
Source: Health Justice. 2020 Jun 12;8:13. doi: 10.1186/s40352-020-00115-5 (PMC7291757; doi:10.1186/s40352-020-00115-5)
Supplement: Supplementary file 1 — Additional file 1: Table S1. Questions used to identify ACEs with qualifying responses. [file 40352_2020_115_MOESM1_ESM.docx]

**Table S1**: Questions used to identify ACEs with qualifying responses

| **ACE questions.** All ACE questions were preceded by the statement “*While you were growing up, before the age of 18...”* | | | |
| --- | --- | --- | --- |
| **ACE** | **Question** |  | **Qualifying responses** |
| *Physical abuse* | How often did a parent or adult in your home ever hit, beat, kick, or physically hurt you in any way? This does not include gentle smacking for punishment? |  | Once; more than once |
| *Verbal abuse* | How often did a parent or adult in your home ever swear at you, insult you, or put you down? |  | More than once |
| *Sexual abuse* | How often did anyone at least 5 years older than you (including adults) ever touch you sexually? |  | Once or more than once to any of the questions |
|  | How often did anyone at least 5 years older than you (including adults) try to make you touch them sexually? |  |  |
|  | How often did anyone at least 5 years older than you (including adults) force you to have any type of sexual intercourse (oral, anal, or vaginal)? |  |  |
| *Parental separation* | Were your parents ever separated or divorced? |  | Yes |
| *Domestic violence* | How often did your parents or adults in your home ever slap, hit, kick, punch, or beat each other up? |  | Once; more than once |
| *Mental illness* | Did you live with anyone who was depressed, mentally ill, or suicidal? |  | Yes |
| *Alcohol abuse* | Did you live with anyone who was a problem drinker or alcoholic? |  | Yes |
| *Drug abuse* | Did you live with anyone who used illegal street drugs or who abused prescription medications? |  | Yes |
| *Incarceration* | Did you live with anyone who served time or was sentenced to serve time in a prison or young offenders' institution? |  | Yes |
| *Physical neglect* | Did your parent/caregiver for long periods of time not provide you with enough food or drink, clean clothes, or a clean and warm place to live when they could have? |  | Yes |
| *Emotional neglect* | Were there times when there was no adult living with you who made you feel loved? |  | More than once |

ACE = adverse childhood experience.
